# Biopolymer-based functional films for packaging applications: A review

**DOI:** 10.3389/fnut.2022.1000116

**Published:** 2022-08-22

**Authors:** Abdullah, Jiyang Cai, Muhammad Adnan Hafeez, Qun Wang, Shahzad Farooq, Qingrong Huang, Wenni Tian, Jie Xiao

**Affiliations:** ^1^Guangdong Provincial Key Laboratory of Functional Food Active Substances, College of Food Sciences, South China Agricultural University, Guangzhou, China; ^2^Department of Allied Health Sciences, Faculty of Allied Health Sciences, Superior University, Lahore, Pakistan; ^3^College of Biosystems Engineering and Food Science, Zhejiang University, Hangzhou, China; ^4^Department of Food Science, Rutgers, The State University of New Jersey, New Brunswick, NJ, United States

**Keywords:** protein-based films, active packaging, intelligent packaging, advanced fabrication strategies, structural and functional properties, food applications

## Abstract

Food packaging is a coordinated system comprising food processing, protection from contamination and adulteration, transportation and storage, and distribution and consumption at optimal cost with a minimum environmental impact to the packed food commodity. Active packaging involves deliberate addition of the functional ingredients either in the film or the package headspace to preserve the food quality, improve safety and nutrition aspects, and enhance the shelf-life. In this review, recent advances in the fabrication of biopolymer-based films, their classification (biodegradable-, active-, and intelligent packaging films), advanced fabrication strategies (composite-, multilayer-, and emulsified films), and special functions induced by the biopolymers to the film matrix (mechanical-, water resistance and gas barrier-, and optical properties, and bioactive compounds reservoir) were briefly discussed. A summary of conclusions and future perspectives of biopolymer-based packaging films as advanced biomaterial in preserving the food quality, improving safety and nutrition aspects, and enhancing shelf-life of the products was proposed.

## Introduction

Food packaging is a coordinated system for the processing of food and its storage, transportation, distribution, retailing, and ultimate consumption by the end user at an optimal cost. It is also characterized as a tool to contain and protect the commodity with a minimum environmental impact (1). In the developed world, packaging industry contributes >2% of the gross national product, and the food packaging represents a half of this share (2). In 2018, global packaging industry has been reported to be worth of $931.1 billions, and predicted to reach $1177.7 billions in 2025. Interestingly, food packaging industry holds the largest portion costed $293 billions in 2018, which would be forecasted to worth of $423.27 billions in 2025 (3, 4).

The basic functions of packaging include protection to the commodity from external contamination and preservation for a longer period by minimizing the effects of external factors (e.g., moisture, heat, light, microorganisms, insects, and dust particles etc.). In addition, it also helps in preventing tampering and enhancing product traceability (5). Besides the provision of these basic functions, modern packaging technologies, i.e., active packaging interacts with the commodity to incorporate functional ingredients that can release or absorb compounds to or from the packaged commodity or the environment surrounding the commodity resulting in increased shelf-life, safety, and quality (6). Intelligent packaging performs functions and communicates with the consumers’ to facilitate in decision making on quality, safety, and shelf-life by monitoring the conditions (internal and external) of the package, and warns about problems by detecting, tracing, sensing, recording, and applying scientific logic (7, 8).

Among the different packaging materials, glass is considered as the best choice owing to its transparency, inertness, and good barrier properties, however, limitations, including poor portability, brittleness, and heaviness are associated with its usage. Similarly, metal and metal sheet lags were regarded as inappropriate materials due to low inertness, transparency, and portability challenges (9). Petroleum-based plastic films (e.g., polyethylene, polyvinyl chloride, and polypropylene) were widely used as packaging material due to easy availability, good mechanical [tensile strength (TS), elongation at break (EAB), and Young’s modulus], and functional properties (permeability to water vapors, oxygen, carbon dioxide, and aromatic compounds) (10). However, serious disposal problems and negative environmental impacts are associated with these plastics, since, such materials are non-biodegradable and also originate from non-renewable resources (11). Moreover, toxic chemical ingredients (flame retardants, pigments, and plasticizers etc.) used in the preparation of plastic packaging material and microplastics may migrate and contaminate the food stuff, resulting in adverse health effects. Furthermore, the microplastics and derived nanoparticles end up in the ocean, and being transformed into persistent organic pollutants that may enter into the food chain by consumption of the marine products (12). Moreover, old fashioned technologies used for the dumping of plastic materials, including landfilling and burning further aggravate the environmental and health risks (13). Regarding the application of petroleum-based films in food preservation, their incapability to incorporate active compounds do not satisfy the demand for achieving antimicrobial and/or antioxidative effects. The above-mentioned situation demands the researchers to fabricate eco-friendly packaging biomaterials from natural and renewable resources with biosafety, biocompatible, and biodegradable characteristics. In this regard, a large amount of agricultural by-products produced every year had the potential to be used in food packaging applications as biodegradable, renewable, and cost-competitive raw material.

The biopolymers (e.g., polysaccharides, proteins, and lipids) can be decomposed into CH_4_, CO_2_, H_2_O, and inorganic compounds mainly by the action of microorganisms (14). A large amount of agricultural by-products produced every year had the potential to be used in food packaging applications as biodegradable, renewable, and cost-competitive raw material (15). Biodegradable polymers possess esters and amide bonds in their chemical structure due to the presence of biodegradable functional groups. During the degradation process of aqueous and enzymatic hydrolysis, these biopolymers turns into shorter and water soluble polymers (16), and decomposed completely without producing any toxic and harmful substances (17). Therefore, application of biopolymers in developing packaging materials is considered highly promising owing to low risk of toxicity or harmful chemicals production, an easy break down into harmless end-products and subsequently become part of the soil (18).

Plant and animal based biopolymers such as polysaccharides, lipids, and proteins as well as different bioplastics produced by microbial synthesis have been used to develop eco-friendly food packaging material with promising potential such as carrier for functional compounds (17–24). The provision of high quality safe food products with a longer shelf-life is another critical issue in packaged food (25). The functional ingredients loaded packaging films provided protection to food stuff from physical, chemical, and biological hazards (5, 6, 26). Moreover, the incorporation of various antimicrobial and antioxidant agents in packaging films offers potent antimicrobial and antioxidant activities (27). For instance, several research studies reported on the use of several metals and their oxides as nanomaterials, including Ag, Cu, CuO, TiO_2_, and ZnO, to form antimicrobial films (25, 28–31). Recently, studies reported the fabrication of several biodegradable films having antifungal properties and subsequently prolonged the shelf-life of the packaged food (32–35). Likewise, different types of antioxidant packaging materials have also been formulated using essential oils, plant extracts, and natural pigments such as curcumin, melanin, and anthocyanins (10, 26, 36, 37).

**FIGURE 1:**
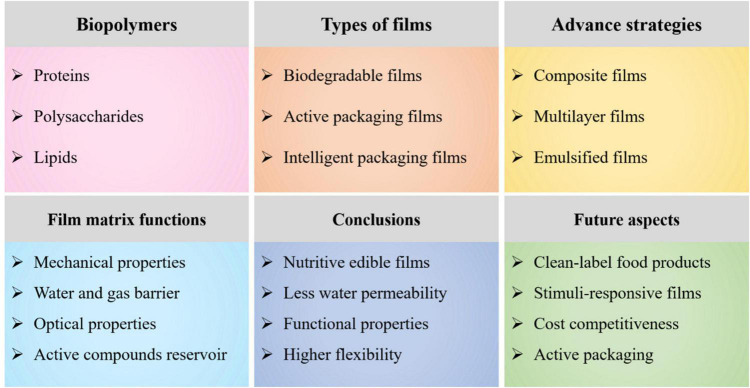
An overview diagram elaborating biopolymer-based films, their classification, advance strategies to fulfill functionality of films, special functions induced by biopolymers to the films matrix, conclusions, and future aspects.

This review highlights the recent advances in the fabrication of biopolymer-based films for food packaging applications (Table 1). The different types of films (biodegradable-, active-, and intelligent packaging films), advanced fabrication strategies (composite-, multilayer-, and emulsified films) to achieve desired functionality of films, and functions induced by biopolymers to the matrix have been comprehensively discussed. This article also provides a detailed understanding of the special functions, including mechanical-, water resistance and gas barrier-, and optical properties, and bioactive compounds reservoir induced due to constitutive nature of the film matrix, i.e., biopolymers and functional ingredients (Figure 1). Current challenges faced in the production of biopolymer-based films and future perspectives emphasizing industrial applications to preserve the product quality, improve safety and nutrition aspects, and enhance shelf-life were also highlighted.

**TABLE 1:** Biopolymer-based functional films for active packaging applications.

Film	Active compound	Key findings	Application	References
Zein/gelatin and polyethylene	Oregano essential oil	Excellent moisture absorption (12.7–21%), water barrier (water vapor transfer rate, 10.3–11.2 gm^–2^ d^–1^), mechanical properties (tensile strength, 18.6–28.3 MPa, elongation at break, 125–191%), significant reduction in the weight loss of fruits, and inhibited bacterial growth (lowest total plate count of 4.9 and 4.3 log CFU/g for longan and strawberry, respectively).	Active packaging	Cai et al. (109)
Zein, gelatin, and paraffin	Oregano essential oil (OEO) and tea polyphenol (TP)	Monolayer and bilayer films containing OEO and TP were developed by tuning the zein/gelatin/paraffin ratio. The obtained film had excellent water barrier ability (lowest water vapor permeability, 0.75 × 10^–10^ gm^–1^ s^–1^ Pa^–1^), and exhibited controlled release for OEO and TP due to the bilayer structure.	Active packaging	Cai et al. (119)
Corn starch and chitosan	–	Improved mechanical strength (tensile strength increased from 4.2 to 6.5 MPa), and water vapor barrier property (water vapor permeability decreased from 21 × 10^–11^ to 3 × 10^–14^ gm^–1^ s^–1^ Pa^–1^).	Food packaging	Fonseca-García et al. (99)
Carrageenan	Titanium dioxide nanotube and copper oxide	Inhibited the growth of *E. coli* (2.5 CFU/mL) and *L. monocytogenes* (6.5 CFU/mL), maintained firmness of bananas (40–60%), and retarded weight loss (2.2%) during 12-days storage.	Active packaging	Ezati et al. (157)
Gelatin	Grapefruit seed extract and titanium dioxide (TiO_2)_	Active ingredients addition increased the film tensile strength (63.4 MPa), stiffness (9.6%), water contact angle (59.3°), and antioxidant activities (DPPH, 7.5–31% and ABTS, 29–57%).	Active packaging	Riahi et al. (23)
Zein and gelatin	Oregano essential oil (OEO) and tea polyphenol (TP)	Optimal tensile strength (14.13 MPa) and one-way moisture barrier properties (2.53 × 10^–10^ at air side, 2.96 × 10^–10^ gm^–1^ s^–1^ Pa^–1^ at bottom side), simultaneous loading of OEO and TP, and higher retention rate and controlled release of OEO and TP.	Active packaging	Chen et al. (100)
Pectin, alginate, and casein	Probiotic (*Enterococcus faecium* Rp1)	Antimicrobial and antioxidant properties, improved structural, optical, and thermal properties, and effective cargo for probiotics.	Active packaging	Namratha et al. (158)
Whey protein isolate/psyllium seed gum	–	Oxygen barrier (permeability decreased from 0.11 × 10^–2^ to 0.06 × 10^–2^ gm^–2^ s^–1^), and improved mechanical strength two times higher than the single whey protein film.	Food packaging	Zhang et al. (159)
Chitosan	Chinese chive root extract	Active ingredients addition (5%) significantly reduced the film water solubility (31.6–18.7%), swelling degree (57.4–40.5%), and water vapor permeability (15.67–7.81 × 10^–11^ gm^–1^ s^–1^ Pa^–1^). Significantly improved antioxidant properties (DPPH, 6.95–47.05% and ABTS, 11.98–57.38%), and antibacterial effects (inhibition zone) against bacterial strains; *B. cereus* (18.79 ± 0.37 mm), *S. aureus* (18.12 ± 0.36 mm), *E. coli* (16.21 ± 0.32), and *S.* Typhimurium (14.91 ± 0.29 mm).	Active packaging	Riaz et al. (79)
Polyvinyl alcohol and gelatin	Amaranthus leaf extract (ALE)	Increased films thickness (100–160 μm), tensile strength (15.8–19.2 MPa), puncture strength (1.7–1.95 N), and antioxidant activity (DPPH, 42.58%). Reduced water solubility (35%) and swelling capacity (43%). Fish and chicken packed in ALE incorporated active film significantly enhanced shelf-life up to 12-days compared to neat-film (3-days).	Active packaging	Kanatt (78)
Carboxymethyl cellulose	Carbon quantum dots (CQD)	CQD addition increased the tensile strength (27.6%), elastic modulus (61.5%), and antioxidant activities (ABTS, 100% and DPPH 88%). CQD incorporated film preserved the appearance, taste, and controlled mold growth on lemon fruits during 21-days storage.	Active packaging	Riahi et al. (160)
Kafirin	Quercetin	Improved water holding capacity (∼85.5%), preserved the sensory quality of cod filets during storage, significant reduction in total microbial content (∼7.2 log_10_ CFU/g), total volatile basic-nitrogen (∼33 mg N 100/g), and thiobarbituric acid reactive substances (∼0.75 mg MDA/kg).	Active packaging	Huang et al. (161)
Zein and chitosan	α-tocopherol	Zein-chitosan-tocopherol (ZCT) films improved the physicochemical properties and enzyme activities of mushrooms during storage at 4°C for 12-days than control, chitosan, and chitosan-zein film. The physiological quality parameters of ZCT vs. control on the 12th day of mushroom preservation were; weight loss (∼28 vs. ∼47%), firmness (∼15 vs. ∼5.5 N), relative leakage rate (∼32 vs. ∼40%), and browning index (∼28 vs. ∼47 BI). The antioxidant capacity in terms polyphenol oxidase, peroxidase, and malondialdehyde contents were significantly decreased in ZCT package (∼0.25 U/kg, ∼0.00 U/kg, and ∼0.2 × 10^6^ mole/L, respectively) than control (∼0.35 U/kg, ∼0.025 U/kg, and ∼0.35 × 10^6^ mole/L, respectively). Regarding enzymatic potential, catalase (0.06 U/kg), superoxide dismutase (0.9 × 10^3^ U/kg), and DPPH radical scavenging activity (∼73%) were significantly increased in ZCT treated mushrooms compared to control.	Active packaging	Zhang et al. (162)
Zein and gelatin	Oregano essential oil (OEO)	Different concentrations of OEO were incorporated in zein/gelatin matrix to form multilayer films which showed a higher retention rate (65%), controlled sustained release in glycerol aqueous food simulant, and inhibited rotting effect and mold formation on strawberries (lowest rotten rate, 20.8%) during 6-days storage at 20°C.	Active packaging	Chen et al. (103)
Potato starch, glycerol, and olive oil	Zein nanoparticles (ZNP)	Olive oil and zein nanoparticles addition improved the film water vapor barrier capacity, and hydrophobicity (water contact angle, 97.98° compared to neat starch film 41.96°). Film color changed to yellowish and offered protection against UV light upon addition of ZNP. Briefly, ZNPs increased the mechanical strength and thermal stability while olive oil showed irreversible behavior. The study revealed starch-zein-olive oil films had promising applications as UV-shielding and water vapor barrier packing materials.	Food packaging	Farajpour et al. (163)
Lentil protein and starch	–	Improved mechanical properties (Young’s modulus, 4.1–22 MPa, stress at break, 2.8–2.4 MPa), and water resistance (water vapor permeability, 2.8 × 10^–10^ to 1.4 × 10^–10^ gm^–1^ s^–1^ Pa^–1^).	Food packaging	Yepes et al. (98)
Zein and gelatin	Tea polyphenol (TP)	Tunable one-way water barrier property (3.00 × 10^–10^ gm^–1^ s^–1^ Pa^–1^ at air side, 1.70 × 10^–10^ gm^–1^ s^–1^ Pa^–1^ at bottom side), prolonged release property (slowed down 0.25-times at the endpoint of release), reduction in weight loss, browning, and bacterial deterioration of kiwi fruits.	Active packaging (kiwi fruit)	Xia et al. (106)
Hydroxypropyl starch and zein	–	Improved mechanical property (tensile strength increased from 14.65 to 17.35 MPa), water resistance (water vapor permeability decreased from 3.38 × 10^–10^ to 2.00 × 10^–10^ gm^–1^ s^–1^ Pa^–1^), transparency, and ultraviolet barrier property.	Food packaging	Chen et al. (107)
Sodium alginate and chitosan	Cinnamon essential oil (CEO)	Improved physical, mechanical, and functional properties (tensile strength increased from 56.36 to 78.58 MPa), sustained release and higher retention rate of CEO (prevented the CEO loss up to 70%), and inhibition of *penicillium* expansion.	Active packaging	Zhang et al. (105)
Starch and polycaprolactone (PCL) or poly (lactic acid) (PLA)	–	Excellent water barrier property (0.145–0.146 gm^–2^ day^–1^), and comparable tensile strength (15–18 MPa for PCL/starch films and 45–56 MPa for PLA/starch films) to plastic films.	Food packaging	Heidemann et al. (108)
Carboxymethyl chitosan nanoparticles and sodium alginate	Zinc oxide (ZnO)	Enhanced mechanical property (tensile strength increased from 16.25 to 24.38 MPa), water vapor resistance (water vapor permeability decreased from 0.49 to 0.21 gm^–2^ h^–1^ kpa^–1^), and antibacterial activity against *S. aureus* (increased from 20% to about 91%) and *E. coli* (increased from 27.7% to about 88%).	Food packaging	Wang et al. (164)
Gelatin, beeswax, and carnauba wax	–	Improved mechanical properties (tensile strength, 0.6–2.3 MPa, and elongation at break, 220–350%), water vapor barrier (4 × 10^–8^ gm^–2^ h^–1^ cm^–2^ Pa^–1^), and antioxidant activity.	Active packaging	Zhang and Simpson (114)
Casein and waxes (beeswax, candelilla, and carnauba waxes)	Potassium sorbate	Reduced water vapor permeability (4.9 × 10^–12^ gm^–1^ s^–1^ Pa^–1^) due to addition of beeswax, and growth inhibition of *E. coli* (zone of inhibition, 8 mm after 20-days of incubation).	Food packaging	Chevalier et al. (165)
Zein	Pomegranate peel extract (PPE)	PPE addition (75 mg) improved the film mechanical properties; higher tensile strength (28.907 MPa), elongation at break (30.458%), and water solubility (18.29%). Enhanced the total phenolic content (∼0.05 mg GA/g) compared to control (∼0.002 mg GA/g), retarded oxidation of fats, preserved the sensory quality of Himalayan cheese, and imparted significant inhibitory effects against pathogens such as *Escherichia coli*, *Pseudomonas perfringens*, *Micrococcus luteus*, *Enterococcus faecalis*, *Staphylococcus aureus*, *Proteus vulgaris*, and *Salmonella* Typhimurium.	Food packaging	Mushtaq et al. (166)
Chitosan	Apricot kernel oil	Increased tensile strength (94%), decreased water vapor transmission rate and water vapor permeability (41%), and improved antioxidant activities (DPPH, 35.3%, and H_2_O_2_, 32.9%). Film effectively inhibited the bacterial growth (*Bacillus subtilis* and *Escherichia coli*) as no viable colony was observed. Likewise, film retarded the fungal (*Rhizopus stolonifer*) growth during a 10-days storage.	Active packaging	Priyadarshi et al. (76)
Poly(vinyl alcohol-co-ethylene)	Gallic acid and umbelliferone	Films effectively inhibited the growth of *Xanthomonas axonopodis* pv. *vesicatoria* CFBP 3274, *Pectobacterium carotovorum* subsp. *Odoriferum* CFBP 1878T, *Erwinia carotovora* subsp. *carotovora* CFBP 2577, and *Botrytis cinerea* CBS 120091. Films antioxidant, thermal, and mechanical (tensile strength and Young’s and elastic moduli) properties remarkably improved due to the addition of bioactive compounds.	Active packaging	Luzi et al. (77)
Kafirin	Citral and quercetin	Citral and quercetin loaded films showed antimicrobial activities against the total viable count on chicken stored at 2 ± 0.5°C for 96 h. The combined effect of citral and quercetin in kafirin film showed a lower value (0.16 mg MDA/kg) of TBARS as compared to control (0.59 mg MDA/kg) and unwrapped filets (0.41 mg MDA/kg).	Food packaging	Giteru et al. (167)
Kafirin and polycaprolactone (PCL)	Carnosic acid (CA)	Kafirin and PCL (1:3) film containing water-soluble drug showed sustained released through a diffusion-controlled manner. Amorphous region of kafirin dominated the release rate, and PCL functioned as the hydrophobic skeleton to maintain the 3D scaffold of the matrix.	Nutraceutical delivery	Xiao et al. (168)
Whey protein isolate and almond or walnut oils	–	Decreased swelling index (47.1–50.5%), water vapor permeability (8.8–13.5 gm^–1^ d^–1^ kPa^–1^), and surface hydrophilicity (contact angle, 36.0–56.4° at air side), and increased oxygen (114–157 cm^3^ μm m^–2^ d^–1^ kPa^–1^) and carbon dioxide permeability (16.9–40.9 cm^3^ μm m^–2^ d^–1^ kPa^–1^).	Food packaging	Galus and Kadziñska (50)
Kafirin and glycerol	*Lactobacillus plantarum* CIDCA 8327, and *Kluyveromyces marxianus* CIDCA 8154	The film thickness, moisture content, and optical properties were same as without the incorporation of microorganisms (*L. plantarum* CIDCA 8327 and *K. marxianus* CIDCA 8154). The viability of *K. marxianus* CIDCA 8154 retained throughout the storage period and observed >90% yeast survival after 30-days storage at 20°C. Inclusion of *K. marxianus* significantly enhanced the survival rate against the sequential acid-bile treatment (36.3%) as compared to their free cells (8.7%).	Probiotics delivery	Piermaria et al. (169)
Zein and fatty acids	(+)-Catechin and lysozyme	Active films showed desired antioxidant activity (81 μmol Trolox/cm^2^), and controlled release properties; slower release rates for loaded compounds, i.e., lysozyme (2- to 8.5-folds) and (+)-catechin (1.6- to 2.9-folds) compared to neat zein film (control).	Active packaging	Arcan and Yemeniciog?lu (153)
Gelatin and olive oil	–	Improved mechanical properties (tensile strength, 24.2 MPa, and elongation at break of 50.5%), and water barrier ability (water vapor permeability, 3.74 × 10^–12^ gm^–1^ s^–1^ Pa^–1^).	Food packaging	Ma et al. (111)
Quinoa protein and chitosan	–	Improved tensile strength (increased from 2.3 to 22.2 MPa), and water barrier (water vapor permeability decreased from 9.4 × 10^–4^ to 3.8 × 10^–4^ gm^–2^ h^–1^ m^–2^ Pa^–1^).	Food packaging	Abugoch et al. (95)

## Classification of films

### Biodegradable packaging films

The edible film and coating terms are referred as synonyms and can be defined as “a primary packaging material that is made up of edible ingredients,” however, they differ in mode of application. Briefly, the edible film is solidified before applying on the packaged commodity while coating is applied in liquid form and subsequently dried (38). Biodegradable films produced from the materials which can be decomposed into the soil after their intended use. In this regard, the use of natural biopolymers in the preparation of edible films has gained significant attention during the last two decades as a safe alternative to petroleum-based packaging materials due to their negative environmental impact and serious disposal problems (39). These polymers are mainly classified into three categories on the basis of their origin and method of production. The first category includes the biopolymers directly extracted from biomass (carbohydrates and proteins), and being widely used in food packaging applications due to their good barrier properties (40, 41). The second category of polymers were synthesized from biobased monomers by classical polymerization, such as polylactic acid (42), and the third category of polymers produced by the actions of microorganisms (43).

In recent years, applications of edible films have rapidly increased in the food industry due to their biodegradable property as well as increasing the shelf-life of packaged products (44–46). Fruits and vegetables based biopolymers have been widely used in the preparation of biodegradable films, and such films successfully preserved the food quality as well as improved the shelf-life and sensory characteristics (46–50). However, it has been reported that the films prepared from these biopolymers exhibited brittleness in their structure ascribed to extensive intermolecular forces which can be inhibited through plasticizer addition, although at the expense of a higher water vapor permeability (51). The addition of a plasticizer accelerates water vapor transmission by enhancing water vapor diffusivity through the polymeric structure by increasing the inter-chain spacing between polymer chains (52, 53).

This section summarizes the preparation and characterization of biodegradable films prepared from different fruits and vegetables such as apple (54, 55), banana (46, 56), guava (57), mango (47, 58), papaya (59, 60), tomato (61, 62), and sugar beetroot etc. (63). Shin et al. (54) developed apple peel powder and carboxymethyl cellulose based biodegradable packaging films by blending these biopolymers along with nanoclay (cloisite Na^+^) using high pressure homogenization technology. The resultant films exhibited good physical, mechanical, and functional properties such as transparency, thickness, EAB, and water vapor permeability ascribed to the formation of cloisite Na^+^ nanocomposite. The authors suggested that developing such novel biodegradable packaging films as a promising practical strategy to transform agricultural biomass into eco-friendly packaging materials with improved physicochemical properties. Shin et al. (55) developed apple peel powder based active edible coating by blending it with carboxymethyl cellulose and tartaric acid as an antimicrobial agent via high pressure homogenization technique. The results indicated that apple peel based active edible coatings proved highly effective in suppressing microbial growth (bacteria, mold, and yeast) and complete inhibition of lipid oxidation of the beef patties. Interestingly, the active coating solution could not affect the sensorial characteristics of the raw and cooked beef patties, thus, active edible coatings could possibly be used to improve microbiological safety, prevent deterioration, and extend shelf-life of food products. Du et al. (61) prepared a tomato puree-based edible film by incorporating allspice, garlic, and oregano essential oils. The obtained film exhibited desired mechanical properties, water resistance ability and optical property as well as showed remarkable antimicrobial activities against *Escherichia coli* O157:H7, *Salmonella enterica*, and *Listeria monocytogenes*. Azeredo et al. (47) developed mango puree-based edible films and employed cellulose nanofibers for nanoreinforcement to improve the mechanical and barrier properties of films. The results indicated that the mango puree-based films showed better TS and water barrier ability after incorporation of cellulose nanofibers that led to the formation of a fibrillar network within the matrix.

### Active packaging films

Modern definition of the active packaging is “any packaging system in which active ingredients have been deliberately added (in/on) either in the packaging material or the package headspace to improve the safety and stability of the packaged products (64, 65).” The basic principles of active packaging rely on the properties of the biopolymers that constitute packaging material and certain active substances (e.g., antimicrobials, antioxidants, phytochemicals, moisture absorbers, and probiotics) embedded inside the biopolymer-based matrix (66–68). The package system has the ability to potentially release desirable active constituents as well as block gases, moisture, or other undesirable compounds (69). Briefly, the active packaging systems can be classified into two major categories depending upon their functionality; (i) emitters that release active substances with desirable activities to bring a positive impact in the package, and (ii) scavengers that reduce undesirable compounds, such as ethylene, carbon dioxide, oxygen, moisture, and off-odors within the packaging environment (6). In this regard, antioxidant-, antibacterial-, antifungal agents, and essential oils have been incorporated into the active films as functional ingredients. Among these active agents, the incorporation of antimicrobial and antioxidant agents into the active packaging films have been recognized as the most promising because they can enhance the shelf-life, preserve quality, and improve safety of products by inhibiting the growth and proliferation of food pathogens (70). Also, several studies have suggested that plant-based polyphenols can be used as a green alternative to synthetic antimicrobials and antioxidants possessing serious health risks (71).

Several bioactive ingredients have been explored to fabricate packaging films that showed potentials by imparting antioxidant (26, 72), antimicrobial (2, 73), antifungal (26, 74), and controlled release properties (11, 75). The bioactive phytochemicals have been reported to be a potential source of potent antioxidants (e.g., polyphenols, flavonoids, tannins), besides improving the antimicrobial and mechanical properties of biopolymer-based functional films (7). Priyadarshi et al. (76) prepared chitosan-based film via solvent casting as active food packaging material by incorporating apricot (*Prunus armeniaca*) kernel essential oil as the functional ingredient. When the chitosan to apricot kernel essential oil ratio was 1:1, the fabricated film showed following improvements: the water vapor barrier capacity increased by 41%, and TS by 94%, however, a proportional increment in the TS value was observed with the increasing oil concentration. Compared to neat chitosan film, the oil incorporated film presented excellent antimicrobial and antioxidant properties by virtue of *N*-methyl-2-pyrrolidone, a strong antimicrobial as well as antioxidant substance present in the oil. Briefly, when the formed film was tested as active food packaging material, it inhibited the growth of *Bacillus subtilis* (Gram-positive) and *E. coli* (Gram-negative) bacteria as well as *Rhizopus stolonifer* (bread mold fungi), indicating antimicrobial potential of film that could be used commercially to enhance the shelf-life of food products. Luzi et al. (77) fabricated poly (vinyl alcohol-co-ethylene) based films by solvent casting and extrusion methods comprising two bioactive compounds, including gallic acid and umbelliferone at 5wt% and 15wt%, respectively. The study results showed that active formulations loaded films successfully inhibited the formation of pathogenic bacterial biofilms (*Xanthomonas axonopodis* pv. *vesicatoria* CFBP3274, *Pectobacterium carotovorum* CFBP 1878T, and *Erwinia carotovora* CFBP 2577), and fungal growth (*Botrytis cinerea* CBS 120091) after 48 h and 7 days incubation, respectively. However, the addition of active ingredients had no significant impact on the films mechanical properties in terms of TS and elastic modulus, indicating films potential for food packaging applications to preserve the quality during processing, transportation, and storage. Kanatt (78) prepared an active packaging film using polyvinyl alcohol (PVA) and gelatin (GE) biopolymers as matrix containing *Amaranthus* leaf extract (ALE). Their results indicated that the film thickness, TS, and puncture strength of extract incorporated films were increased from 100 to 160 μm, 15.8 to 19.2 MPa, and 1.7–1.95 N, respectively, as compared to films without ALE. Moreover, the water solubility and swelling capacity of extract containing films were found less by 35.03 and 42.84% compared to the neat film. The films containing extract presented excellent antibacterial properties: the inhibition zones recorded for *Bacillus cereus*, *Staphylococcus aureus*, *E. coli*, and *Pseudomonas fluorescens* were 33 ± 2 mm, 30 ± 1 mm, 26 ± 1 mm, and 25 ± 1 mm, respectively, while neat films exhibited no antibacterial effects. In addition, the antioxidant activity evaluated by DPPH scavenging activity was significantly higher in extract containing film (42.58%) as compared to neat film (2.2%). Besides, when active film was applied on fish and chicken during chilled storage, it enhanced the shelf-life up to 12-days, while the neat film had a shelf-life of only 3-days. The lipid oxidation of fish and chicken stored in PVA-GE-based film increased to 2.89 and 1.25 mg MDA/kg, respectively after 3-days, while extract incorporated film (PVA-GE-ALE) showed an increase of 1.3 and 1.07 mg MDA/kg even after 12-days of storage. The study suggested that the PVA-GE-ALE films had better physical, mechanical, antibacterial, and antioxidant properties, as well as preserved the fish and chicken by retarding oxidative changes during chilled storage.

Riaz et al. (79) developed chitosan-based films via solvent casting method by incorporating Chinese chive root extract (1, 3, and 5% w/w) containing phenolic compounds. The resultant films properties were significantly improved as the water solubility, swelling degree, and water vapor permeability decreased from 31.6 to 18.7%, 57.4 to 40.5%, and 15.67 to 7.81 × 10^–11^ gm^–1^ s^–1^ Pa^–1^, respectively. Moreover, the antioxidant activities in terms of DPPH and ABTS free radicals scavenging capacity significantly increased from 6.95 to 47.05% and 11.98 to 57.38%, respectively. The color of the films became more yellowish and darker when the amount of Chinese chive root extract increased up to 5 percent. Regarding antimicrobial activity, the films containing 5% Chinese chive root extract had promising inhibitory effects against different bacteria, including *B. cereus*, *S. aureus*, *E. coli*, and *Salmonella* Typhimurium with zones of inhibition 18.79 ± 0.37 mm, 18.12 ± 0.36 mm, 16.21 ± 0.32 mm, and 14.91 ± 0.29 mm, respectively. The study’s findings showed that functional films exhibited promising antioxidant and antimicrobial properties that could possibly be used as a bio-composite packaging film for food packaging applications at commercial level.

### Intelligent packaging films

**FIGURE 2:**
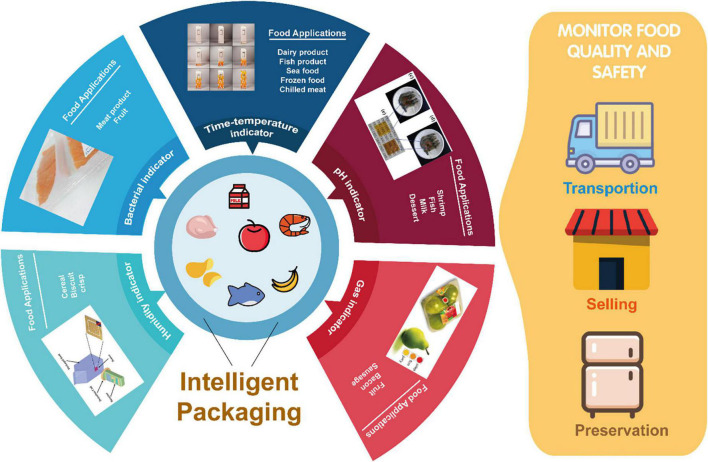
Emerging industrial applications of biopolymer-based intelligent packaging films being employed for monitoring the quality of food such as fruits, meat, and dairy products.

Intelligent packaging is defined as “a coordinated system of food packaging that enables the consumers’ to obtain information about the quality in the food supply chain by monitoring the storage conditions of the packaged food” (80). This process is carried out with the aid of different sensors and indicators linked to the packaging system or by means of colorimetric changes (81, 82). The sensors identify, record, and transmit the quality changes and product information, while indicators monitor time, temperature, and pH (83). Figure 2 indicates several intelligent packaging systems such as gas, pH, time, temperature, and humidity sensors, being incorporated into or printed onto the food packaging materials in order to monitor the real time quality of the packaged food (84). In recent years, studies have also reported the development of biosensors being employed for the detection of pathogenic bacteria (85, 86).

Among the intelligent packaging systems, pH indicators are the most promising packaging systems comprising a pH sensitive solid base and a dye (87). The dyes used in this packaging system are extracted from fruits and vegetables. A change in pH occurs when a certain food starts its deterioration process, which indicates the quality changes with the change in pH (7). Ezati and Rhim (88) fabricated a chitosan-based packaging film through casting method with the inclusion of pH responsive alizarin and evaluated as intelligent packaging applications on fish. The alizarin incorporated chitosan film was proved to be sensitive to the ammonia vapors and a change in color from yellow to purple was observed with the change in pH from 4 to 10. The results suggested that the prepared composite film could be used to detect the onset of fish spoilage by monitoring color change from khaki to light brown as the pH of the packaged fish changed. Li et al. (89) formulated chitosan (CS) based colorimetric pH indicator as intelligent packaging film by incorporating different concentrations (10, 30, and 50%) of purple tomato anthocyanins (PTA) by casting method to evaluate milk and fish freshness/spoilage. The potential application of PTA-CS composite film as intelligent packaging to monitor food spoilage and freshness was confirmed, as the film with 10% PTA responded to the progressive spoilage of milk or fish with color changes (from pink → light purple → bright green → light green → light yellow at pH = 3, pH = 5, pH = 7, pH = 9, and pH = 11, respectively).

Pacquit et al. (90) formulated a smart packaging film to detect fish spoilage using bromocresol green (BCG) (sodium salt) as pH sensitive dye which is then spin-coated at 1,000, 2,000, and 3,000 rpm onto optically clear polyethylene terephthalate (PET) substrate disks. The results exhibited that with the increase in spin rate, the thickness of the sensor decreased (2.57, 1.36, and 1.01 μm at a spin rate of 1,000, 2,000, and 3,000 rpm, respectively). The thickest sensors, coated at 1,000 rpm reflected higher signal strength than others and a more intense color change was visible with the naked eye. Keeping in view this clear color indication of the said sensor, the authors further utilized this sensor for fish spoilage trials. The results showed that during fish spoilage trial, reflectance colorimeter was unable to detect any color change in the first 14 h. However, a significant increase in reflectance was observed at 16–18 h. As the sensor changed color from yellow to green and then blue at 43 h, and no further color change was observed. Conclusively, it was suggested that the prepared pH sensitive dye responded to the amines secreted from the fish with the change in color. Zhai et al. (91) developed a pH sensitive intelligent packaging film using gellan gum, gelatin, and red radish anthocyanins extract to monitor fish spoilage. The composite film showed an orange red to yellow color change in the pH range of 2–12. The authors explained that the fabricated composite film showed visible color changes during fish spoilage, besides its use as pH indicators as the intelligent packaging systems.

Besides, the pH and color indicators, recently food freshness indicators (FFI) and time-temperature indicators (TTI) have paved their way in the field of intelligent packaging system with the potential of rapid response to stored food quality and temperature changes through color changes. In this regard, electrospinning technology have been used in the recent years to fabricate FFI/TTI intelligent packaging owing to its higher porosity, higher molecular orientation, and higher level of versatility (92). Maftoonazad and Ramaswamy (93) formulated PVA based electrospun nanofibers with the incorporation of red cabbage extract (RCE) to assess its potential for the packaging and real time monitoring of fresh date fruits (rutab) during storage. The results of the colorimetric analysis explained that an intense color change was recorded in various pH values with the increase in anthocyanins concentration from 10 to 30%. The results suggested that the indicator color changed from blue to violet and then purple from the start of the storage time to the spoilage step, respectively. This change in color occurred due to the increased concentration of CO_2_ in the packaging headspace. In another study, Xu et al. (94) used electrospinning technique to develop an oxygen sensor to monitor the respiration and freshness of grapes. The authors used *tris* (4,7-diphenyl-1,10-phenanthroline) ruthenium (II) dichloride (RuDPP) as an oxygen-sensitive probe along with silane-functionalized carbon dots (SiCDs) as oxygen-insensitive luminophore in the said oxygen sensitive nanofibrous membranes. The luminescent emission intensity of the designed nanofiber mats increased with the decreased oxygen level and increased respiration rate. The fabricated FFI exhibited different colors starting from brilliant blue to purple and light red fluorescent on days 6 and 7 successively, indicating that the oxygen in the package had been exhausted and resulted in deterioration of grapes quality.

## Advanced strategies to achieve functionality of films

### Composite films

The formation of a composite film involves blending of two or more matrixes to form a monolayer film, and it is a well-used strategy to integrate the advantages and functionalities of different matrixes. The biopolymers being used in the production of composite films include proteins, polysaccharides, and lipids. In this section, only the hydrocolloids biopolymers (proteins and polysaccharides) were discussed, since they have emerged as a promising strategy to improve the mechanical and water barrier properties due to the molecular entanglements among the proteins and polysaccharides molecules (95–97). The blending of hydrocolloids with lipids will be introduced in the section of emulsified films.

Yepes et al. (98) prepared a lentil protein-starch composite film by casting and extrusion/thermo-compression methods and concluded that the presence of protein improved the mechanical properties including Young’s modulus from 4.1 to 22 MPa, and stress at break from 2.8 to 2.4 MPa as well as water resistance decreased in terms of water vapor permeability from 2.8 × 10^–10^ to 1.4 × 10^–10^ gm^–1^ s^–1^ Pa^–1^. Recently, a new formulation of corn starch-chitosan with the addition of copolymer pluronic F127 was evaluated, and the resultant film showed improved TS (increased from 4.2 to 6.5 MPa), and water vapor barrier properties (water vapor permeability decreased from 21 × 10^–11^ to 3 × 10^–14^ gm^–1^ s^–1^ Pa^–1^) due to increased hydrophobicity of the film matrix ascribed to the addition of pluronic F127 (99). Our research group, Chen et al. (100) fabricated a zein-gelatin composite film with customized phase separation through the bench casting method. By tuning the mass ratio of zein to gelatin, different phase separation behaviors of the biopolymers were thoroughly observed. The hydrophobic essential oil and hydrophilic tea polyphenol (TP) can be simultaneously loaded into the composite film due to the phase separation of zein and gelatin matrixes. In addition, the controlled phase separation behaviors further modulated the proper mechanical property (TS of 14.13 MPa and EAB of 32.82%), and achieved the one-way moisture barrier properties (2.53 × 10^–10^ gm^–1^ s^–1^ Pa^–1^ at air side, 2.96 × 10^–10^ gm^–1^ s^–1^ Pa^–1^ at bottom side). Recently, Sood and Saini (101) designed a biopolymer film comprising red pomelo peel pectin, casein, and egg albumin, and the results showed that the incorporation of pectin endowed a smoother and more compact film structure. When the red pomelo peel pectin, casein, and egg albumin with ratio of 50:50:0 and 50:25:25 were used, the enhanced water resistance ability (water vapor permeability of 0.81–0.83 × 10^–12^ gm^–1^ s^–1^ Pa^–1^) and higher TS (4.03–4.10 MPa) were observed in the resulting films.

### Multilayer films

Multilayer film consisting of two or more biopolymers, is one of the most effective approach to fabricate a biopolymer-based film with enhanced performance. Multilayer film integrates the advantages of different layers of the film made from different materials to improve the mechanical and functional properties, including TS, water barrier property, and simultaneous loading as well as controlled release of the bioactive compounds. Recently, interest in the industrial application of multilayer approach in the formation of films has attracted tremendous attention of researchers (102–104).

Zhang et al. (105) developed a multilayer film by solvent casting, comprising chitosan and sodium alginate biopolymers containing cinnamon essential oil (CEO) as the antimicrobial agent. The results showed that the multilayer film not only improved the mechanical properties (TS, 78.58 MPa) compared to the film with a single layer (TS, 56.36 MPa), but also the release and retention parameters of the CEO (prevented the essential oil loss of up to 70%) were greatly improved. In our previous work, Xia et al. (106) fabricated multilayer film with the stacking order of zein as an outer layer, hybrid zein/gelatin as middle layer, and gelatin as an inner layer using the bench casting method. Briefly, the zein layer performed as a hydrophobic layer against the moisture migration and the gelatin layer acted as a hydrophilic layer for moisture absorption. Thus, the one-way water barrier property (3.00 × 10^–10^ gm^–1^ s^–1^ Pa^–1^ at air side, 1.70 × 10^–10^ gm^–1^ s^–1^ Pa^–1^ at bottom side) was gained by employing the multilayer approach. In addition, the TP with concentration gradient was then incorporated into the middle and inner layers, and later on successfully released in a sustained release manner. The TP-loaded film effectively retarded weight loss, rapid browning, and bacterial growth during the preservation of freshly cut fruits. We then, prepared a hydroxypropyl starch/zein bilayer film through a two-step solvent casting method, wherein the hydroxypropyl starch and zein solutions casting led to the formation of hydroxypropyl starch and zein layers (107). The results indicated significant improvements in film water resistance ability (water vapor permeability decreased from 3.38 × 10^–10^ to 2.00 × 10^–10^ gm^–1^ s^–1^ Pa^–1^) and mechanical property (TS increased from 14.65 to 17.35 MPa). Moreover, an adhesion interface was developed between the two layers due to the conjunction of starch and zein matrixes through hydrogen bonding. In addition, the antimicrobial or antioxidant bioactive compounds with various polarities can be simultaneously incorporated into the multilayer structures aiming controlled/sustainable release.

Another strategy to meet the market demand in developing efficient active films is to laminate the biopolymer film with the plastic substrate, which simultaneously combined the advantages of biobased film for loading and controlled release of natural active compounds and the excellent water resistance and mechanical properties of plastic ones. The development of biopolymer-plastic multilayer film is a feasible and promising approach in solving the difficulties involved in the production of biopolymer-based active films at the industrial scale. Heidemann et al. (108) fabricated multilayer films by the combination of starch with two synthetic biodegradable polymers including polycaprolactone (PCL) or poly (lactic acid) (PLA), using the cold plasma technique to improve the compatibility between two layers. The results showed that the moisture barrier property of the multilayer films was strongly improved (water vapor transfer rate, 0.145–0.146 gm^–2^ day^–1^) compared to the pure starch films (water vapor transfer rate, 2.8 gm^–2^ day^–1^), while the TS of multilayer films (15–18 MPa for PCL/starch films and 45–56 MPa for PLA/starch films) were comparable to that of PCL (19 MPa) or PLA (58 MPa) films. In another work, we combined the zein/gelatin biopolymers with commercial polyethylene (PE) film using an industrial dry laminator, and successfully prepared a novel biopolymer-coated active packaging (109). The obtained multilayer film exerted functionalities, including antimicrobial activity (lowest TPC of 4.9 and 4.3 log CFU/g for longan and strawberry, respectively), moisture absorption (12.7–21%), excellent moisture barrier (water vapor transfer rate, 10.3–11.2 gm^–2^ d^–1^), and mechanical properties (TS, 18.6–28.3 MPa, and EAB, 125–191%). Despite the advantages of improving the mechanical and functional attributes of biopolymer-based film, the production of multilayer film requires more casting time and high energy consumption compared to monolayer film. Nevertheless, the poor recyclability and degradability of plastic layer in multilayer film had adverse effects on the environment.

### Emulsified films

**FIGURE 3:**
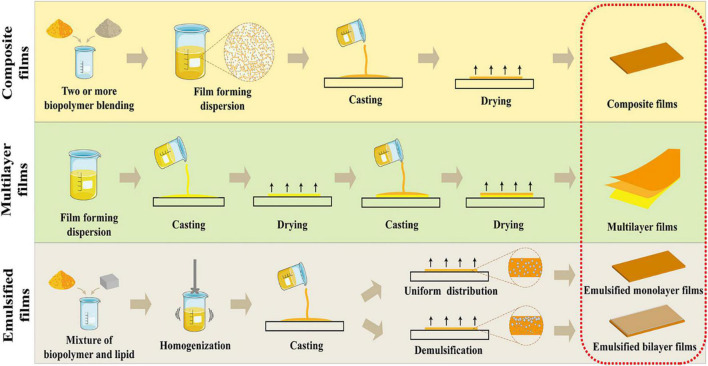
Schematic illustration of the advanced film forming strategies to fulfill functionality of biopolymer-based films with improved mechanical and functional properties for food packaging applications.

The formation of an emulsified film involves dispersing lipids with biopolymers (polysaccharides or proteins hydrocolloids) to form a film with a monolayer or a bilayer structure (Figure 3). The biopolymer-based film originating from polysaccharides and proteins possesses a good film-forming property but poor moisture resistance ability. In comparison with the hydrocolloids, lipids, the naturally hydrophobic biopolymers present excellent moisture resistance ability, but their non-polymeric character limits their ability to form a free-standing film. One practice to circumvent this inherent drawback is the addition of hydrophobic substances such as lipid with the hydrophilic protein or polysaccharide biopolymers, by either laminating the lipid onto the film to form the bilayer film or emulsifying the lipid within the hydrocolloid matrix to develop emulsified film. In the formation of bilayer film, lipids were usually cast as the second layer over the protein or polysaccharide layer, which required several steps (two castings and two dryings) during the film casting process that consumed more energy and organic solvent. And bilayer films were easily subjected to delamination and crack over time (110, 111). As for emulsified film with monolayer structure, lipids were evenly distributed in the hydrocolloid matrix, and resultant films exhibited high productivity and low cost for industrial productions due to the one-step casting technique. However, their water resistance ability was much lower than the bilayer films. The emulsification process is necessary for the preparation of emulsified monolayer films. And the homogenization deeply affected the droplet size and distribution, which play an important role in the moisture barrier property of obtained emulsified monolayer films. In general, the smaller droplet size and narrower distribution lead to lower moisture permeability. Flores et al. (112) developed a chitosan-carvacrol monolayer emulsified film and investigated the effect of different homogenization process on the droplet size of oil phase and on the corresponding physicochemical properties. The results showed that high-pressure homogenization led to smaller droplet size and homogeneous distribution of carvacrol oil phase within the film matrix. As a consequence, the higher water barrier ability was obtained (about 2.10 × 10^–10^ gm^–1^ s^–1^ Pa^–1^), compared the film prepared by rotor-stator method (about 2.5 × 10^–10^ gm^–1^ s^–1^ Pa^–1^). Xue et al. (113) produced the essential oil loaded monolayer emulsified film, where the soy protein isolates-gum acacia used as the continuous phase and essential oils including oregano, lemon or grapefruit incorporated as dispersed phase. Among the formed films, the grapefruit essential oil-incorporated film exhibited the highest water barrier ability (1.66 × 10^–10^ gm^–1^ s^–1^ Pa^–1^) attributed to smaller droplet size of grapefruit essential oil and a more uniform oil distribution within the film matrix. In addition, the lipid polarity and fraction also play a key role in the functional property of emulsified monolayer film. Conclusively, the moisture barrier property of films was improved as the lipid fraction increased, but obtained films became more brittle and less stretchable (114).

In recent years, the preparation of emulsified bilayer films using the “emulsion technique” has been proposed for the integration of advantages over bilayer films and monolayer emulsified film. The “emulsion technique” is a one-step approach that involves dispersion of the lipids within the hydrocolloid biopolymers solution forming an emulsion and inducing the demulsification of the emulsion during the film casting process, which resulted in the floating of lipids to form a successive layer. Compared to the emulsified monolayer films, the emulsified bilayer films exhibited a better moisture resistance ability. The moisture barrier properties of bilayer film depends on whether the resulting film had a successive lipid layer, indicating excellent water resistance capacity. Fabra et al. (115) fabricated a sodium caseinate emulsified film containing oleic and stearic acids, respectively. The results showed that when the oleic acid was loaded in the film, a bilayer-like structure developed and oleic acid was homogeneously distributed in the film matrix. And accordingly, the stearic acid-containing film showed a better water resistance ability (16.0 × 10^–10^ gm^–1^ s^–1^ Pa^–1^) than the oleic acid (24.1 × 10^–10^ gm^–1^ s^–1^ Pa^–1^), suggesting that the bilayer structure provided greater perpendicular resistance to the mass transfer of water molecules.

Numerous studies attempted to regulate the emulsion destabilization to form a successive lipid layer by tuning temperature, homogenization conditions, drying rate, viscosity, lipids concentration, interactions between components of films, and emulsifier application (115–117). Zhang et al. (118) developed an agar/maltodextrin-beeswax emulsified film and investigated the effect of drying temperatures and homogenization conditions on the fabrication of bilayer films. The results showed that the pseudo-bilayer structure was obtained at higher drying temperature and lower homogenization intensity, and exhibited excellent water resistance ability (2.18 × 10^–12^ gm^–1^ s^–1^ Pa^–1^) and mechanical properties (TS of 20 MPa and EAB of 38.9%). Recently, we fabricated an emulsified film comprising zein, gelatin, and paraffin, and found that the distribution of lipids within zein/gelatin matrix could be controlled to form either the emulsified monolayer or bilayer films, via tuning the ratio of zein and gelatin (119). When the matrix was gelatin-dominated, the successive or partial bilayer films developed, while the monolayer films produced in zein-dominated matrix.

### Functions induced by biopolymers to the film matrix

**FIGURE 4:**
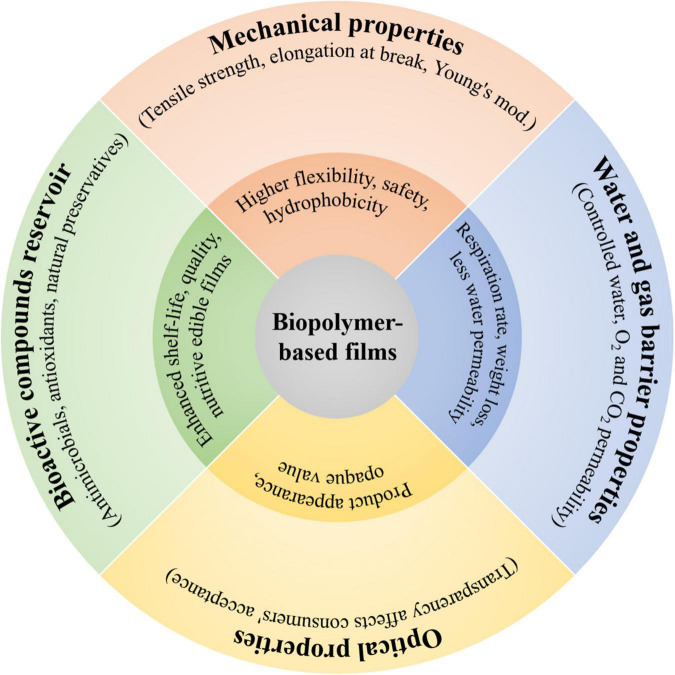
Special functions induced by biopolymers to the film matrix, including mechanical-, water and gas barrier-, and optical properties as well as bioactive compounds reservoir.

The functional properties induced by biopolymers to the films include mechanical strength, water resistance and gas permeability, transparency, and as bioactive compounds reservoir (Figure 4). These functional properties are of key importance to achieve desired performance and functionalities such as integrity of film matrix, controlling the mass transfer between food and external environment, enhancing the sensory attributes, and delivering the active compounds to interact with food. Therefore, assessing and rationally controlling the functional properties during fabrication of biopolymer-based films have great significance for a range of food packaging applications.

### Mechanical properties

The mechanical properties, including TS, EAB, and Young’s modulus are the important properties for films being used in food packaging. The appropriate mechanical properties ensure functional material capacity to safeguard the food commodities during their transportation such as handling, storage, and distribution until consumption. Mechanical properties mainly depend on the composition and chemical nature of the biomaterials being used in the formation of films. Among the protein-, polysaccharide-, and lipid-based biopolymers, the lipids could not form a free-standing film, while the protein- and polysaccharide-based polymers exhibit excellent film-forming properties (120). However, the pure biopolymer-based films showed poor mechanical properties compared to the plastic polymers such as PE, PP, and PET.

In recent years, emerging trends that have been used to improve the mechanical properties of biopolymer-based films include not only the strategies to construct composite- and multilayer films discussed in Advanced Strategies to Achieve Functionality of Films, but also other methods such as the addition of nanoparticles or nanomaterials and the incorporation of nanofillers. In this regard, several research works used nanoparticles for reinforcement in the fabrication of biopolymer-based films. Gahruie et al. (121) selected basil seed gum as the film matrix, wherein the *Zataria multiflora* essential oil nanoemulsion was immobilized within the basil seed gum matrix. The results indicated that increase in the nanoemulsion concentration in basil seed gum matrix enhanced the mechanical property of films (TS increased from 19.74 to 34.64 MPa, EAB increased from 21.55 to 39.54 MPa). Besides, incorporation of nanofiller has also been widely reported to reinforce the biopolymer-based films. Chaichi et al. (122) added crystalline nanocellulose with three concentrations (2, 5, and 7% w/w) into edible pectin film and evaluated the reinforcement effects of nanocellulose on the mechanical properties of pectin film. The results indicated that the addition of nanocellulose simultaneously improved TS and EAB of composite film, and also improved the composite film water barrier properties. At 5% concentration of nanocellulose in the film-forming solution, the results showed that the film TS increased up to 84% and water vapor permeability decreased by 40%.

The incorporation of additives such as plasticizers and antimicrobial agents, may change the mechanical properties of biopolymer-based films, since the addition of these compounds led to changes in the intermolecular arrangement or cross-linking in the biopolymer molecules (123). Since, natural hydrocolloids exhibit brittle mechanical properties, therefore, plasticizers are usually added to the biopolymer-based film-forming solutions to improve flexibility in terms of increase in EAB value. Plasticizers are a class of small molecules with average molecular weights of between 300 and 600 Da that could reduce the tension of deformation, hardness, density of the biopolymer, and increase the chain flexibility of biopolymer by breaking the polymer-polymer interactions, and creating the polymer-plasticizer interactions (52, 53, 124). As a result, the addition of plasticizer endows the flexibility and softness to biopolymer-based films. In the formation of biopolymer-based films, food-grade plasticizers are usually used such as glycerol, polyethylene glycol (PEG), and sorbitol etc. Literature review showed that the incorporation of plasticizers increased the EAB and decreased the TS of the biopolymers (125). However, different studies had reported different results depending on the type and character of plasticizers and biopolymers, and their potential interactions. Laohakunjit and Noomhorm, (126) reported that glycerol and sorbitol made starch film softer (EAB increased from 1% to 12–24%), while the TS reduced from 9 to 1–3 MPa. In comparison, PEG 400 plasticized films showed poor mechanical properties, while the biopolymer plasticized by sugars such as fructose, maltose, sucrose, and D-allulose showed preferable mechanical properties including TS and EAB than the glycerol, sorbitol, and glycols (125, 127, 128).

### Water resistance

Water resistance of films plays an important role in preventing deteriorative reactions in foods. Therefore, it is the most widely studied property of films. The biomacromolecules naturally exhibit a strong hydrophilic character, and this drawback limits their applications as the packaging material for food with high water activity. Generally, the efforts to improve the water resistance ability of the biopolymers are mainly through emulsified film strategies, i.e., addition of lipids and waxes as moisture barriers. Besides, other methods such as forming the multilayer- and composite films to improve the water barrier properties have also been practiced (discussed in Advanced Strategies to Achieve Functionality of Films). This phenomenon was mainly attributed to the fact that the inclusion of lipids increases the hydrophobic nature of films and the tortuosity for water transfer into the matrix.

The strategy of combining lipids with biopolymers in the preparation of multilayer or emulsified films has emerged as an important technique to improve the water resistance of the resultant films. For bilayer composite films, the polysaccharide or protein film layer is coated or dipped with the melted lipid or wax solution, forming the second lipid layer. Regarding the emulsified films, the lipid or wax is firstly dispersed in the polysaccharide or protein solution in the form of emulsion, and entrapped as the dispersed phase in the biopolymer matrix after casting. The bilayer film showed superior water resistance properties than the emulsified film, due to the perpendicular resistance of the successive lipid layer to water molecules (116, 117). However, the bilayer films were easily subjected to delamination and crack over time, and this technique required a two-step casting process that need more energy consumption. For the emulsified monolayer films, the effect of lipids and waxes on the improvement of water resistance is highly dependent on droplets size and distribution in the biopolymer matrix (129). The smaller droplet size or more narrow distribution of dispersed lipids, the better water resistance ability was observed in the emulsified films. Therefore, the emulsion destabilization phenomena such as flocculation, creaming and coalescence should be finely controlled during the casting process. Ma et al. (111) investigated the effect of homogenization conditions of film-forming solution on the lipid droplet size and physical property of gelatin-olive oil emulsified film. The results showed that microfluidizer with higher energy input produced narrow-sized lipids particles in the film, and subsequently improved the water barrier ability (water vapor permeability of 3.74 × 10^–12^ gm^–1^ s^–1^ Pa^–1^) and mechanical properties (TS of 24.2 MPa and EAB of 50.5%) Gul et al. (130) incorporated clove essential oil into the hazelnut meal protein using the ultrasound homogenization to obtain nanoemulsion film. The results showed that the ultrasound treatment enabled the film microstructure more homogeneous and reduced the droplet size of clove essential oil dispersing in film matrix. The water vapor permeability of obtained film significantly decreased to 2.8 g mm m^–2^ h^–1^ KPa^–1^, compared to control film (5.9 g mm m^–2^ h^–1^ KPa^–1^).

### Gas barrier property

The gas concentration is highly correlated to the food quality including the aroma, flavor, color, texture, and nutrition. Therefore, the gas barrier properties of biopolymer-based films should be emphasized during the preservation of foods. Compared to the majority of plastic films, one advantage of the biopolymer-based films is their preferable gas barrier property, since the hydrocolloids polymers (proteins and polysaccharides) are naturally polar that impart good barrier properties to gases including O_2_ and CO_2_. The addition of lipids and hydrophobic antimicrobials (essential oils) have been reported to increase the O_2_ and CO_2_ permeability (109, 131, 132). However, the opposite results were also observed after incorporation of some oils, for example, carvacrol (133), citral, and lemongrass essential oils (134).

The gas barrier property of biopolymer-based films should be suitable for preserved food. For example, during the preservation of fruits and vegetables, suitable gas permeability of biopolymer-based films reduced the gas concentration inside the packaging and decreased the respiration rate of fruits and vegetables to a bearable value, which decreased weight loss and nutrition loss. However, excessively low gas barrier of biopolymer-based films would cause anaerobic respiration, leading to the production of off-flavor and deterioration of texture of the fruits and vegetables. Therefore, precise control of the gas permeability for the biopolymer films is quite important to maintain food quality (135). Sun et al. (136) developed a novel biopolymer-based film containing methylcellulose (continuous phase) and cellulose nanocrystals (dispersed phase). The obtained film increased the CO_2_/O_2_ selectivity (permeability ratio of CO_2_ to O_2_) due to the ability of film matrix to discriminate the molecular size of gases, and different affinity of polymers matrix toward gases, which was beneficial in decreasing the respiration rate of fruits. Zhou et al. (137) introduced a novel biomimetic strategy for the fabrication of plant leaf-mimetic films with controllable gas permeation. Wherein, the poly (L-lactic acid) (PLLA) or chitosan porous microspheres with nanosized pore structures exhibited different permeability to O_2_ and CO_2_, specifically, designed as the gas “switches” or “stomata” in a shellac film. The results showed that by incorporating different amounts of porous microspheres according to the respiratory rate of fruits, the O_2_, CO_2_, and H_2_O permeability and CO_2_/O_2_ selectivity of the novel film could be controlled, which showed exceptional preservation performance on five selected model fruits with different respiratory metabolisms.

### Optical property

The optical property usually characterized by the transparency value is an important parameter for biopolymer-based film, since it affects the packed product appearance thus highly linked with the consumers’ acceptance. Pure hydrocolloid-based biopolymer usually exhibits high transparency, which is associated with the homogeneity of biopolymer matrix (138). After the addition of lipids, the transparency of biopolymer-based film decreased associated with the content of lipids added in the film-forming solution (139–143). The presence of lipids creates heterogeneous networks within the hydrocolloid matrix, thereby resulting in the light scattering effect and lowering of transparency. Briefly, the influence of the lipid phase on the optical property of the biopolymer-based film usually depends on the lipid fraction, droplet size, and distribution with the matrix. Numerous studies have corroborated that larger-sized lipid droplets evoked more light scattering effect than smaller-ones, and consequently increased the opaque value (111, 115, 130).

Jiménez et al. (144) studied the effect of four saturated and unsaturated fatty acids on the hydroxypropyl-methylcellulose (HPMC) films. The results exhibited that upon the addition of saturated fatty acids, the transparency of films decreased with the increase in the size of lipid aggregates. Besides, other additives such as antimicrobials and antioxidants can also influence the optical property of biopolymer-based films. Malik and Mitra (145) developed an active packaging by incorporating the zinc oxide (ZnO) nanoparticles as the antimicrobial agent into HPMC films. The results showed that when ZnO nanoparticles were added into HPMC matrix, the transparency of films significantly decreased since the incorporation of ZnO nanoparticles with white color increased the light scattering effect in film matrix. Acevedo-fani et al. (146) developed an alginate-based film by incorporating essential oil-loaded nanoemulsion to protect the essential oils degradation. The three essential oils were selected, including thyme (TH-EO), lemongrass (LG-EO), and sage (SG-EO). The edible film containing LG-EO showed the highest opaque value compared to other films, due to its coarse surface caused by the floating of oil droplets to film surface during the film drying process.

### Bioactive compounds reservoir

Bioactive compounds application in active food packaging have rapidly increased due to their desirable preservative effects on the food quality compared to synthetic active compounds as well as due to their toxicological safety and exceptional functional (antimicrobial and antioxidant) properties. Recently, this practice has gained great interest in the field of active food packaging due to health-promoting benefits in addition to the preservative effects. The ability of biopolymer-based films for loading and controlled release of active compounds is an unparalleled superiority attracting current research interests compared to the plastic films with poor compatibility to bioactive compounds. Attributing to this trait, the bioactive compounds, including antimicrobials, antioxidants, flavors, natural pigments, and even pharmaceutical or nutraceutical ingredients can be efficiently loaded into biopolymer-based films to design active food packaging and cosmetics biomaterials.

The encapsulation and controlled release properties of active compounds from the films had great significance for the preservation of food. For example, the rapid release of active compounds would result in limited long-time antimicrobial effects for food preservations, while the slow release of active compounds may not reach the minimum inhibitory concentration value. Therefore, to rationally design the controlled release property of functional films has attracted the attention of researchers working in academia and industries to fulfill the consumers’ demand for “clean-label” food products. Extensive efforts have been made to achieve the controlled release of bioactive compounds via immobilization into nanoemulsions (147), Pickering emulsions and emulsion gels (148–150), nanoliposomes (151), β-cyclodextrin (152), nanosphere (153), and using multilayer technique (105).

Yuan et al. (154) prepared a novel starch-based film loaded with thyme oil microemulsions or microcapsules. The results showed that the employment of microemulsions or microcapsules significantly decreased the release rate of thyme oil. The antibacterial activity of film containing microemulsions was stronger than the film containing microcapsules. The film containing microcapsules dramatically increased the shelf-life of chilled meat (14-days), compared to the blank control and the PE film with shelf-life of 6- and 10-days, respectively. Peretto et al. (155) developed a strawberry puree based edible film as carrier for loading carvacrol and methyl cinnamate. The results showed that the controlled release of the active compounds from the films contributed to retain the firmness and brightness, and also increased the total phenolics content and antioxidant activity of strawberries at the end time (10-days) of preservation, compared to the untreated strawberries. Esmaeili et al. (156) investigated the effect of chitosan and whey protein films loaded with nanoencapsulated garlic essential oil aimed to extend the shelf-life of refrigerated vacuum-packed sausages, wherein the nanoencapsulation enhanced the controlled release property of essential oil. The chitosan films loaded with nanoencapsulated garlic essential oil presented the promising preservative effects on sausages up to 50-days, measured in terms of the peroxide value (0.37 meq/kg lipid), thiobarbituric acid reactive substances (0.47 mg malondialdehyde/kg), and aerobic plate count (3.69 log CFU/g).

## Conclusion and future perspectives

Biopolymer-based active packaging films have attracted increasing attention for packaging applications to meet consumers’ demand of “all natural” food products and the industrial drive of providing “clean-label” products. In recent years, applications of biopolymer-based films have rapidly increased for industrial applications due to their attractive properties, including nutrition value, improved mechanical and functional properties, biocompatibility and biodegradability, and source renewability and eco-friendly nature advantages. Moreover, biopolymers fabrication potential has boosted their applications in the food industry as packaging biomaterial that ideally suited to enable the incorporation of active compounds (e.g., emitters and scavengers) being used to preserve the food quality with enhanced shelf-life. The plant-derived bioactive ingredients, including polyphenols and essential oils not only inhibit and/or delay microbial spoilage, but possess biological properties such as antioxidant-, anti- inflammatory-, and anticancer activities.

Major challenges involved in the development of biopolymer-based films are their poor water resistance and mechanical properties. Though many approaches have been used to improve their final properties, however the obtained films properties were not comparable with their synthetic non-degradable or degradable counterparts. Therefore, significant technical breakthroughs are needed to improve the physicochemical, mechanical, and functional properties of biopolymer-based films in the future. Of course, it is also appealing to pave the way for their alternative applications, for instance, the poor water resistance and mechanical features may be desirable for some specific applications, when the films are supposed to be orally consumed with the packed food (e.g., packaging of nuts, candies, and snacks etc.) or to be dissolved upon cooking. Another obstacle that limits the applications of biopolymer-based films is the relatively high cost of raw materials. Feasible solutions to commercialize the biopolymer-based film products requires combined efforts from various aspects in terms of research endeavors in lowering the cost of raw materials, the government polices such as subsidies to promote biopolymer-based films usage, investment for film-production equipment, and elimination of manufacturer resistance. Moreover, public and private organizations should do joint ventures to increase the market appeal of biopolymer-based packaging films due to their preferable sensory attributes, nutritional, antioxidant, and antimicrobial properties.

Also, more attention should be paid to their potential toxicity and allergic reactions. To be specific, protein-based polymers (casein and gliadin) and polysaccharide-based polymers (starch), may trigger allergic reactions, therefore, *in vitro* and *in vivo* studies should be designed to address the biosafety issues. Moreover, the profound interest in functional films as active packaging biomaterial encapsulating bioactive ingredients, more work is needed to evaluate their pharmacokinetics, including bio-accessibility, digestion, cell absorption, biochemical transformations, and excretion etc. These aspects should be considered by manufactures to achieve the commercialization success for biopolymer-based packaging films.

## Author contributions

Abdullah and JX: conceptualization. Abdullah, MAH, and JC: writing—original draft preparation. SF, QW, QH, and JX: writing—review and editing. JX: supervision and funding acquisition. All the authors have read, revised, and agreed to manuscript publication.
